# Serological markers to measure recent changes in malaria at population level in Cambodia

**DOI:** 10.1186/s12936-016-1576-z

**Published:** 2016-11-04

**Authors:** Karen Kerkhof, Vincent Sluydts, Laura Willen, Saorin Kim, Lydie Canier, Somony Heng, Takafumi Tsuboi, Tho Sochantha, Siv Sovannaroth, Didier Ménard, Marc Coosemans, Lies Durnez

**Affiliations:** 1 Department of Biomedical Sciences, Institute of Tropical Medicine, Antwerp, Belgium; 2 Department of Biomedical Sciences, University of Antwerp, Antwerp, Belgium; 3 Department of Biology, University of Antwerp, Antwerp, Belgium; 4 Molecular Epidemiology Unit, Institut Pasteur du Cambodge, Phnom Penh, Cambodia; 5 National Centre for Parasitology, Entomology and Malaria Control, Phnom Penh, Cambodia; 6 Division of Malaria Research, Proteo-Science Center, Ehime University, Matsuyama, Japan

**Keywords:** Malaria, Serological markers, Malaria transmission, Half life

## Abstract

**Background:**

Serological markers for exposure to different Plasmodium species have recently been used in multiplex immunoassays based on the Luminex technology. However, interpretation of the assay results requires consideration of the half-life of specific antibodies against these markers. Therefore, the aim of the present study was to document the half-life of malaria specific serological makers, as well as assessing the sensitivity of these markers to pick up recent changes in malaria exposure.

**Methods:**

A recently developed multiplex immunoassay was used to measure the intensity of antibody (Ab) responses against 19 different Plasmodium specific antigens, covering different human malaria parasites and two vector saliva antigens. Therefore, 8439 blood samples from five cross-sectional surveys in Ratanakiri, Cambodia, were analysed. These involve a random selection from two selected surveys, and an additional set of blood samples of individuals that were randomly re-sampled three, four or five times. A generalized estimating equation model and linear regression models were fitted on log transformed antibody intensity data.

**Results:**

Results showed that most (17/21) Ab-responses are higher in PCR positive than PCR negative individuals. Furthermore, these antibody-responses follow the same upward trend within each age group. Estimation of the half-lives showed differences between serological markers that reflect short- (seasonal) and long-term (year round) transmission trends. Ab levels declined significantly together with a decrease of PCR prevalence in a group of malaria endemic villages.

**Conclusion:**

For *Plasmodium falciparum*, antibodies against LSA3.RE, GLURP and Pf.GLURP.R2 are most likely to be a reflexion of recent (range from 6 to 8 months) exposure in the Mekong Subregion. PvEBP is the only *Plasmodium vivax* Ag responding reasonably well, in spite of an estimated Ab half-life of more than 1 year. The use of Ab intensity data rather dichotomizing the continuous Ab-titre data (positive vs negative) will lead to an improved approach for serological surveillance.

**Electronic supplementary material:**

The online version of this article (doi:10.1186/s12936-016-1576-z) contains supplementary material, which is available to authorized users.

## Background

Despite a considerable decline, the malaria burden in the Greater Mekong Subregion is still high and has a major impact on public health, especially in some specific regions within countries [1]. With 68,000 malaria cases in 2013 and an annual parasite incidence rate of 4.6 per 1000 persons, malaria is the main single-cause infectious disease in Cambodia [2]. From 2000 to 2015, Cambodia has achieved a reduction of >75% in malaria case incidence [3], resulting in very low and heterogeneous malaria transmission clustered in hotspots and hotpops [2, 4, 5]. When aiming for malaria elimination, these focused areas of low malaria transmission pose considerable challenges for epidemiological surveillance and evaluation of control and elimination measures [6].

In malaria elimination areas, detection and surveillance of persisting malaria transmission is key for focussing interventions [7]. Traditional techniques for determining malaria transmission intensity include entomological inoculation rates (EIR) and parasite prevalence (PP) estimates [8]. However, in low transmission areas, the EIR and PP lack sensitivity, due to very low numbers of parasite-positive samples, both in mosquitoes and humans [9, 10].

Alternatively, serology is believed to be more informative in obtaining epidemiological information in malaria control programmes. In particular, serological markers can be used as a proxy for *Plasmodium* transmission intensity in low endemic areas [11] where parasite carriage is reduced and vector populations persevere [12]. More specifically this corresponds to the measurement of the force of infection (FOI), reflecting a rate at which susceptible individuals acquire an infection per year in a given area [9]. Where the EIR and PP mainly focus on the presence or absence of the infection [11] serology focuses on antibodies (Abs) that remain in the blood longer than the parasite. This is profitable in measuring host-parasite contact, and may provide information on recent or past malaria exposure, but only in case the half-lives of the Abs are known [11, 13, 14].

From an immunological point of view, partial protective immunity to malaria is built up after recurrent infections, typically over a period of several years of exposure. Hence, different types of memory are expected based on the frequency of short- (several months) and long-lived plasma cells (years) [15]. In areas of seasonal malaria transmission, it is assumed that short-lived plasma cells appear early in life and long-lived plasma cells later on [16], which can be used to understand the measurements of Ab half-lives [16]. It is important to take into account the different parasite life stages (skin, liver and early blood stages) as well [17]. Producing Abs against sporozoites or merozoites is challenging, due to the short time that sporozoites need to reach and penetrate liver cells, and for merozoites to reinvade erythrocytes. Strong Ab-responses are expressed against early blood stage Ags, which can be more useful in estimating cumulative exposure [12, 17]. Even though it is difficult to determine the half-life of Abs, some studies have already studied the Ab persistence [18–20].

**Fig. 1:**
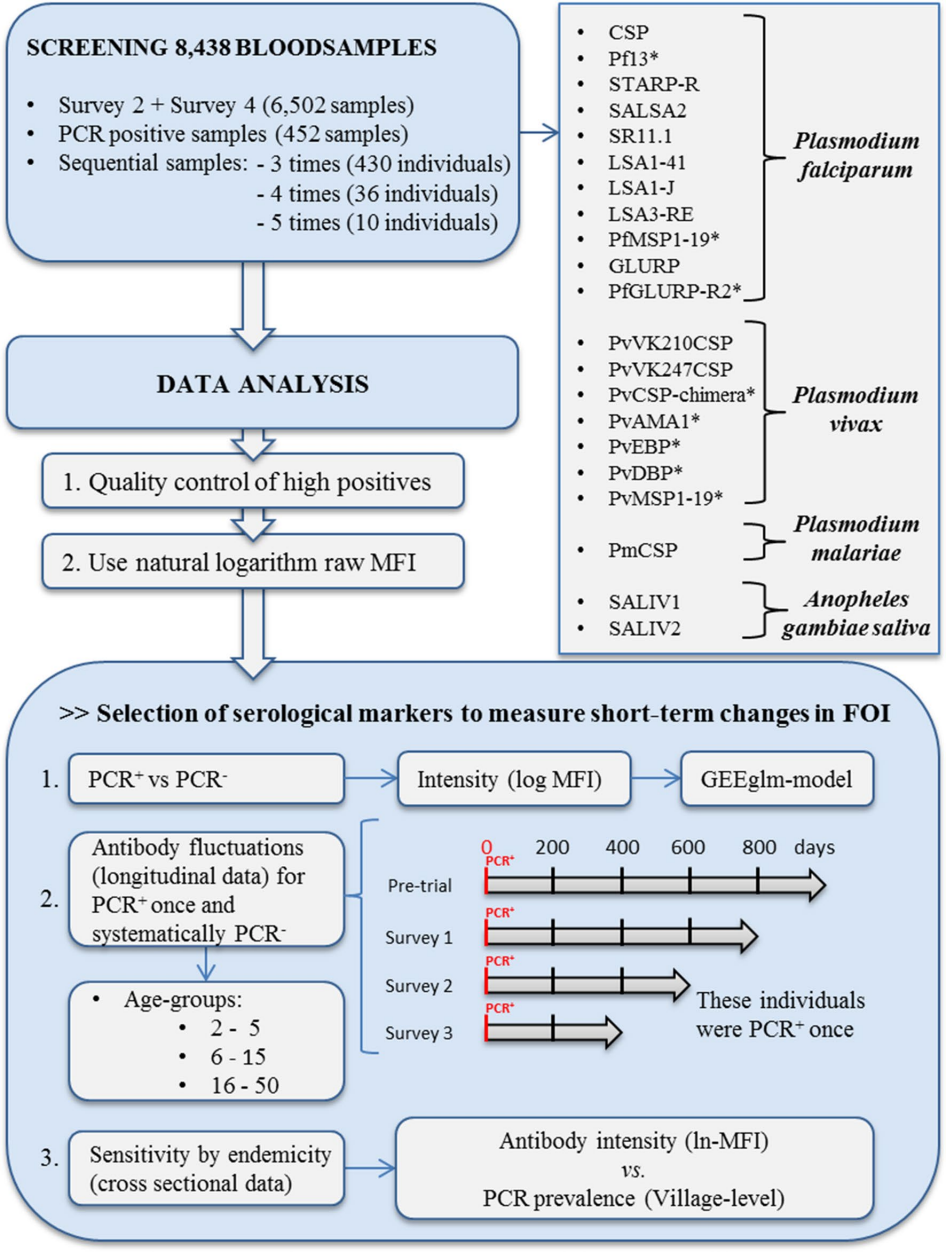
Overview of the study, indicating the antigens used in each step

The aim of the current study is to document the half-life of IgG-Abs against 21 Ags from specific malaria parasites (*Plasmodium falciparum, Plasmodium vivax, Plasmodium malariae*) and saliva of vectors as well as assessing the sensitivity of the markers to pick up recent changes in malaria exposure. Methods to estimate the half-life of serological markers are still in its infancy, therefore, to reach this goal, three different approaches [13, 21] were applied directly on continuous Ab intensity values (Fig. 1) instead of using (arbitrary) thresholds to distinguish positives from negatives [22]. First, a comparison of the Ab-intensity between PCR-positive and PCR-negative samples was performed. Secondly, the half-life of the serological markers was documented by analysing sequential samples. Thirdly, the Ab-responses were examined in relation to the PCR endemicity. As there is no golden standard, the use of multiple approaches will improve the robustness of the results. As such, serological markers that perform best for all three approaches are considered to be most promising in reflecting recent exposure.

## Methods

### Malaria transmission patterns in the study area

Ratanakiri is the highest malaria endemic province of Cambodia. Malaria transmission occurs during the rainy season, between April/May and October/November. Over the years a major decline in malaria transmission had been observed in this province: In 2000, the recorded annual parasite incidence in Ratanakiri was 11/1000 inhabitants, whereas [23, 24] in 2012 an average incidence of 3.1/1000 inhabitants were reported [4]. The observed decline is attributed to the performance of the National Malaria Control Programme (high long-lasting insecticidal net coverage and improved case management) but also to environmental changes (deforestation) impacting the main vector *(Anopheles dirus)* [2].

### Samples

Serum samples used for this study were collected during the cluster randomized trial organized within the MalaResT project, NCT01663831, that aimed to evaluate the use of topical repellents as added control measure to long-lasting insecticidal nets for prevention of malaria [4, 5] in Ratanakiri province, Cambodia. The project took place in the 98 most endemic clusters in Ratanakiri (88 single villages and 25 neighbouring villages grouped into 10 clusters) [25]. These 98 clusters were split into two arms, a control (long-lasting insecticidal nets) and an intervention arm (long-lasting insecticidal nets and the topical repellent picaridin). During 2 consecutive years (2012/2013) individuals were sampled by collecting two drops of blood on filter paper through a finger prick. To obtain baseline information on malaria prevalence at cluster level, the first survey (a pre-trial study) was conducted February 2012 (PCR prevalence 6.34%) [4, 25]. Four additional cross-sectional surveys were performed at the start of the rainy season in April/May (surveys 1, 2012 and 3, 2013) and 6 month later in October/November (surveys 2, 2012 and 4, 2013) with a PCR prevalence between 3 and 4% [25]. No differences in PCR prevalence were observed between the two study arms. PCR positive persons received a 3-day treatment with dihydroartemisinin plus piperaquine immediately following the national treatment guidelines [5, 25]. The immunoassay was performed on a total of 8438 out of 26,929 blood spot samples. Of this total, 6502 samples were randomly selected from survey 2 (3264 out of 4996 samples) and survey 4 (3238 out of 5431 samples). In addition, a total of 452 samples from the pre-trial and all four trial surveys were analysed as they were PCR positive for *Plasmodium* parasites. Furthermore, all samples of individuals that were randomly re-sampled three (430 individuals), four (36 individuals) or five times (10 individuals) during the entire MalaResT project were also included (Fig. 1). For the immunoassay blood spot filter papers were prepared by punching one disc of 6 mm (diameter), and eluted overnight in 160 μL of PBS-TBN [PBS-1% BSA-0.15% Tween, pH 7.4, Sigma-Aldrich (dilution 1:40)]. Just before use in the immunoassay, the eluted samples were diluted to 1:200 in PBS-CR, as previously described [26].

### Antigens

The selection of Ags was based on their availability and tested in a previous study that focused on the implementation of the multiplex bead-based immunoassay [26]. The current assay includes eleven *Plasmodium* specific peptides, and eight *Plasmodium* specific recombinant proteins. These 19 Ags cover most stages of the life cycle of the parasite in the host (Table 1; Fig. 1). In addition, two peptides specific for the *Anopheles gambiae* saliva protein gSG6 (salivary gland 6) were included. All peptides were chemically synthesized with an added N-terminal cysteine residue and BSA (bovine serum albumin, Sigma-Aldrich, St. Louis, USA) [27] by GeneCust Europe (Dudelange, Luxembourg). The recombinant proteins were produced as described in Table 1.

**Table 1:** Overview of the antigens (peptides and recombinant proteins) used in this study

Antigens	Sequence (N-terminal to C-terminal)	g/mol	Life-cycle stages	*Plasmodium* species	Peptide or recombinant protein	Ref.
CSP	NANPNANPNANPNANPNVDPNVDPC	2257.67	Sporozoite	*P. falciparum*	Peptide	[27]
Pf13	C-terminal His-tag produced in *E. coli*		Sporozoite	*P. falciparum*	Recombinant protein	[69, 70]
STARP.R	STDNNNTKTISTDNNNTKTIC	2299.42	Sporozoite and liver stage	*P. falciparum*	Peptide	[27, 71]
SALSA2	NGKDDVKEEKKTNEKKDDGKTDKVQEKVLEKSPKC	4019.52	Sporozoite and liver stage	*P. falciparum*	Peptide	[27, 71]
SR11.1	EEVVEELIEEVIPEELVLC	2213.50	Sporozoite and liver stage	*P. falciparum*	Peptide	[27, 71]
LSA1.41	LAKEKLQEQQSDLEQERLAKEKLQEQQSDLEQERLAKEKE KLQCERRAKEKLQEQQSDLEQRKADTKKC	5297.97	Liver stage	*P. falciparum*	Peptide	[27, 72, 73]
LSA1.J	ERRAKEKLQEQQSDLEQRKADTKKC	3046.43	Liver stage	*P. falciparum*	Peptide	[27, 71, 73]
LSA3.RE	VESVAPSVEESVAPSVEESVAENVEESVC	2991.20	Liver stage	*P. falciparum*	Peptide	[27, 71]
Pf.MSP1.19	Glutathione S-transferase (GST) fusion protein. C-terminal expressed in *E. coli*		Merozoite	*P. falciparum*	Recombinant protein	[38, 40]
GLURP	EDKNEKGQHEIVEVEEILC	2241.47	Trophozoite	*P. falciparum*	Peptide	[27, 71, 74]
Pf.GLURP.R2	C-terminal produced in *E.coli*		Trophozoite	*P. falciparum*	Recombinant protein	[27, 71, 75]
PvVK210.CSP	DGQPAGDRAAGQPAGDRADGQPAGDRADGQPAGC	3206.30	Sporozoite	*P. vivax*	Peptide	[71, 76, 77]
PvVK247.CSP	ANGAGNQPGANGAGNQPGANGAGNQPGANG AGNC	2905.95	Sporozoite	*P. vivax*	Peptide	[71, 76, 77]
PvCSP (chimera)	Soluble His-tag protein expressed in a wheat-germ cell free expression system		Sporozoite	*P. vivax*	Recombinant protein	[76, 77]
PvAMA1			Merozoite	*P. vivax*	Recombinant protein	[78, 79]
PvEBP			Merozoite	*P. vivax*	Recombinant protein	[80, 81]
PvDBP			Merozoite	*P. vivax*	Recombinant protein	[81]
Pv.MSP1.19	C-terminal produced in the baculovirus expression system		Merozoite	*P. vivax*	Recombinant protein	[38, 40, 48]
PmCSP	GNAAGNAAGNDAGNAAGNAAGNAAGNAAGNAAC	2358.37	Sporozoite	*P. malariae*	Peptide	[71]
SALIV1	EKVWVDRDNVYCGHLDCTRVATFC	2830.22	Salivary gland proteins	*An gambiae*	Peptide	[27, 82]
SALIV2	ATFKGERFCTLCDTRHFCECKETREPLC	3324.84	Salivary gland proteins	*An gambiae*	Peptide	[27, 82]

Ags are organized according to the *Plasmodium* species and the life-cycle stages in the human host

### Covalent coupling of antigens to the beads/microspheres

Each Ag was covalently coupled at a concentration of 4 μg Ag/10^6^ beads to 35 × 10^6^ paramagnetic beads/beadset (MagPlex microspheres, Luminex Corp., Austin, TX, USA) [26]. BSA (Sigma-Aldrich, St Louis, USA) was coupled to an additional set of beads to serve as a background control. All beads and the BSA were mixed to prepare a microsphere working mixture. This mixture was then aliquoted and stored at 4 °C in portions of 500 μl per tube [26].

### Bead-based immunoassay

The immunoassay was conducted as previously described [26]. First, a 500 μl aliquot of the microsphere working mixture was diluted to a final volume of 5000 μl (1:10) with a concentration of 1000 beads/Ag/well. From this microsphere working mixture 25 μl was added in each well of a 96-well plate followed by 50 μl of diluted serum sample (1:200) [26]. As a control for the immunoassay, a pool of negative sera (non-exposed European sera) was added to each plate in duplicate at a dilution of 1:100 in PBS-CR. A pool of positive sera containing sera from four individuals tested positive for *P. falciparum* and two for *P. vivax* [26] was added to each plate in duplicate at dilutions 1:100, 1:400 and 1:1600. For the washing steps PBS-TBN was used, and 100 μl/well of secondary Ab (R-phycoerythrin^+^-conjugated AffiniPure F(ab’)_2_ fragment of goat anti-human IgG, Jackson Immuno Research Laboratories) at a dilution of 1:500 was added [26]. All plates were analysed in a random order to minimize a bias. In a final step, beads were resuspended in 100 μl of 5% PBS-BSA, pH 7.4. In total, 216 plates were analysed and read by the MAGPIX^®^ system with a minimum amount of 400 beads per spectral address. Results were represented as the median fluorescent intensity (MFI—Additional file 1) [26].

### Statistical analysis

A scheme of the data analysis can be found in Fig. 1. Raw data were processed and analysed in R version 3.1.0. [28]. To assure the validity of the results of each plate a quality control was performed. Results were corrected for background signal by substracting the signal obtained with BSA-coupled beads (MFI_BSA_) from the median value of the Ag-coupled beads (MFI_Ag_), defined by ∆MFI = MFI_Ag_−MFI_BSA_ [26]. The MFI-values of the high positive control pool samples and the percentage positivity ($\frac{\Delta MFI Low \, positive \, control (Ag1)}{\Delta MFI High\, positive\, control (Ag1)}$ × 100%) from the low positive control (50% value of the high positive control) pool samples were plotted in Levey Jenning Charts. The high positive (100% value) is determined per Ag and based on the 1:100, 1:1400 and 1:1600 serum dilutions. The plates with samples outside the range of −2SD and +2SD were rejected and repeated (Additional file 2) [26].

Further differences between duplicate samples were explored using a quantile regression on an MA-plot using R package *‘OutlierD’* [29]. M is the difference between the duplicate samples and A is the average of the duplicate samples [30, 31]. Samples that fell outside the lower 25% and upper 25% quantiles of this MA-plot were rejected (Additional file 4). Hereafter, the means of the duplicates were calculated per sample of each Ag. All analyses were carried out on the natural logarithm (ln) of the raw MFI in accordance with Helb et al. [13].

As age influences Ab-responses [11, 32], age categories were created based on the work of Kusi et al. [8]. To explore whether the age categories defined were al biologically relevant in the study region, the (ln) MFI values were plotted against age (Additional file 5). Moreover, to account for multiple comparisons Bonferroni corrections were applied to each of the following approaches [33, 34].

Towards selecting markers that pick up contact with the parasite (recent of not recent), ln(MFI) values of PCR positive and PCR negative samples were compared per *Plasmodium* species. Generalized estimating equation (GEE) models were used (geeglm function in the R package ‘*geepack’* [35–37]), taking into account the within-cluster correlations that might exist due to the sampling design (village level clustering) [38]. The model also takes into account PCR positivity and age as factors allowing estimation of “population-averaged” effects [38, 39]. Different GEE-models were compared through ANOVA (a P value < 0.05 was considered significant) tests to select the most appropriate model per Ag: (1) a null model (intercept-only model), (2) a model with only PCR prevalence as a dependent variable, (3) a model with only age groups as a dependent variable, (4) a model with PCR prevalence and age groups as dependent variables (without interaction and (5) with interaction).

To estimate the half-life of the Abs after effective treatment of malaria, the presence of Abs in the same individuals (642 persons) followed up over time (±600 days) was examined [40]. Therefore, only individuals that were PCR positive in at least one survey were selected and the time of PCR positivity (and effective treatment) was taken as time point zero. This allows assessing the dynamics of the Abs against different Ags. Three linear models were compared per Ag through ANOVA tests: (1) a model with time since infection as dependent variable, (2) a model with time since infection and age groups as dependent variable (without interaction and (3) with interaction). Based on the slope (=λ) of the selected model, the half-life was estimated per serological marker (${\text{t}}_{1/2} = \frac{{ - { \ln }(2)}}{\lambda }$) [8, 41]. The 95% confidence intervals (95% CI) were estimated using the *‘confint’* function in R [28]. This analysis was performed on the systematically parasite negative individuals as well.

For determining the association between Ab-levels at population level and PCR-prevalence of asymptomatic *Plasmodium* infections, the ln(MFI) values were plotted against PCR prevalence at cluster level. Therefore, forest plots (multiplot function in the R package *‘grid’* [28] and forest plot function in R package *‘ggplot2’* [42]) were created on the cluster prevalence data from survey 2 and 4 per *Plasmodium* species. For *P. falciparum* and *P. vivax* this was performed on mono infections and for the *An. gambiae* saliva proteins on all infections (regardless of *Plasmodium* species). Each cluster was examined twice, once for survey 2 and once for survey 4. Then, the prevalence data were ordered from low to high prevalence (Additional file 7) and split into different groups representing low, medium and high prevalence (cut2 function in R package *‘Hmisc’* [43]). For each species, groups of clusters that showed an overlap between medium and high-level PCR prevalence were excluded, allowing a clear distinction between three groups (high-, medium- and low-level PCR prevalence). Thereafter, ln(MFI) values were age-adjusted as differences in Ab-levels can be found between different age categories. Finally, linear models taking into account cluster within survey as random effect (lmer function in R package *‘lme4’* [44]) were compared through ANOVA tests: (1) a null model (intercept-only model), (2) a model with PCR prevalence as a dependent variable, (3) a model with PCR prevalence and age as dependent variables (without interaction and (4) with interaction). The Incidence Rate Ratio (IRR) is corresponding to a ratio of Ab-levels estimated directly with the R-software.

## Results

Although PmCSP was included in the battery of Ags, the results for PmCSP are not shown as the MFI signal (mean MFI < 600) did not surpass the background signal for the BSA.

### Quality control

A total of 8654 field blood samples were analysed distributed over 108 96-well plates. Each plate was analysed in duplicate. After quality control, out of the 216 plates 30 plates were repeated (Additional file 2). The control samples fell within the linear range of the assay (Additional file 3). Samples were checked for uniformity across the measuring range of the assay by looking at the between plate variation of the samples (>96%). Thereafter, based on the quantile regression (Additional file 4), between 10 and 17% of the duplicate samples depending on the Ag were not consistent with each other and were excluded from the dataset. When determining the age categories, samples from individuals above 50 years were excluded, due to large variations in MFI values and a limited number of available samples (Additional file 5).

### Comparison of MFI values between PCR positive and PCR negative samples

**Fig. 2:**
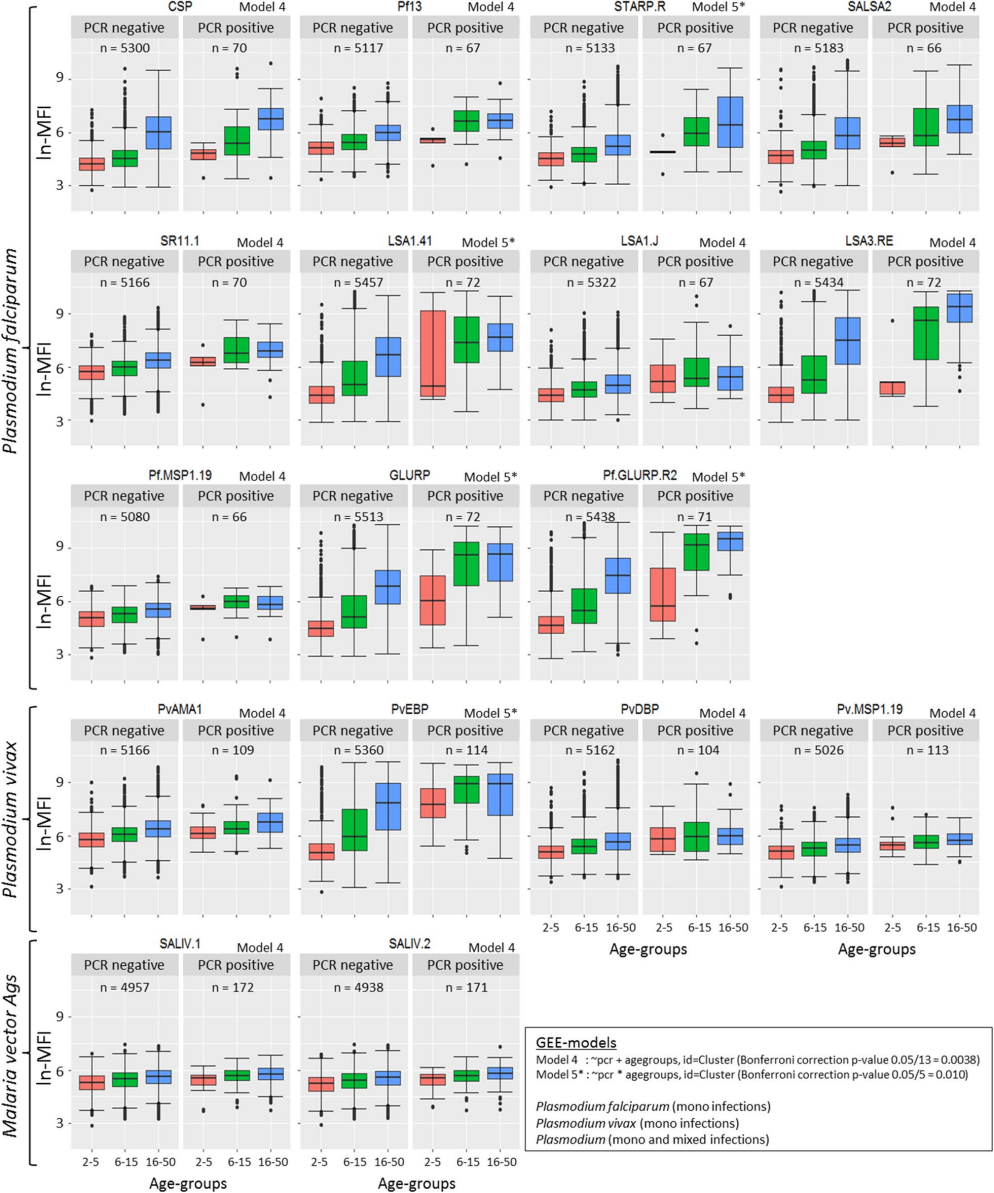
Antibody responses to *Plasmodium* antigens, stratified by age and the presence or absence of malaria infection. Individuals were divided into three age groups (1–5, 6–15 and 16–50) to explore the relation with age. Previous obtained PCR results [4, 5] were used to determine the presence and absence of the *Plasmodium* infection. Boxplots represent the medians, interquartile ranges and *error bars* show 95% confidence intervals. *Circles* represent outlier values. Generalized estimating equation (GEE) models were conducted taking into account Cluster as a random effect. Ags with non-significant results were not included in the figure

For 17 out of 20 Ags, the Ab levels were higher in PCR positive as compared to PCR negative blood samples (P < 0.0025, Bonferroni correction for 20 different tests) (Fig. 2). However, Ags PvVK210.CSP (P = 0.842), PvVK247.CSP (P = 0.557) and PvCSP (P = 0.009) did not show significant differences between the Ab levels within the age groups. As such, these three Ags were omitted from the figure. For 12 out of 20 Ags (CSP, Pf13, SALSA2, SR11.1, LSA1.J, LSA3.RE, Pf.MSP1.19, PvAMA1, PvDBP, Pv.MSP1.19, SALIV1 and SALIV2) there w0as no interaction between PCR status and age group, meaning that PCR-positive persons have significantly higher Ab levels for 12 Ags than PCR-negative persons (Fig. 2), within all age groups (model 4, P < 0.0038).

For five other Ags the model that includes the interaction between PCR-positivity and the age groups was selected as the best model, showing that there is a significant difference in MFI values between PCR positive and PCR negative samples (P < 0.01, Bonferroni correction for five tests), but that the magnitude of the difference depends on the age group. These Ags can be divided into two different groups. First PvEBP shows a significant difference (P < 0.01) between MFI values of PCR positive and PCR negative persons only in the younger age groups (2–5 and 6–15 years old), whereas for STARP.R, LSA1.41, GLURP and Pf.GLURP.R2 the differences in MFI values between PCR-positive and PCR-negative persons are significant (P < 0.01) in the older age groups (6–15 and 16–50 years old) only.

### Ab-responses decline with days since infection, depending on age and serological marker

**Fig. 3:**
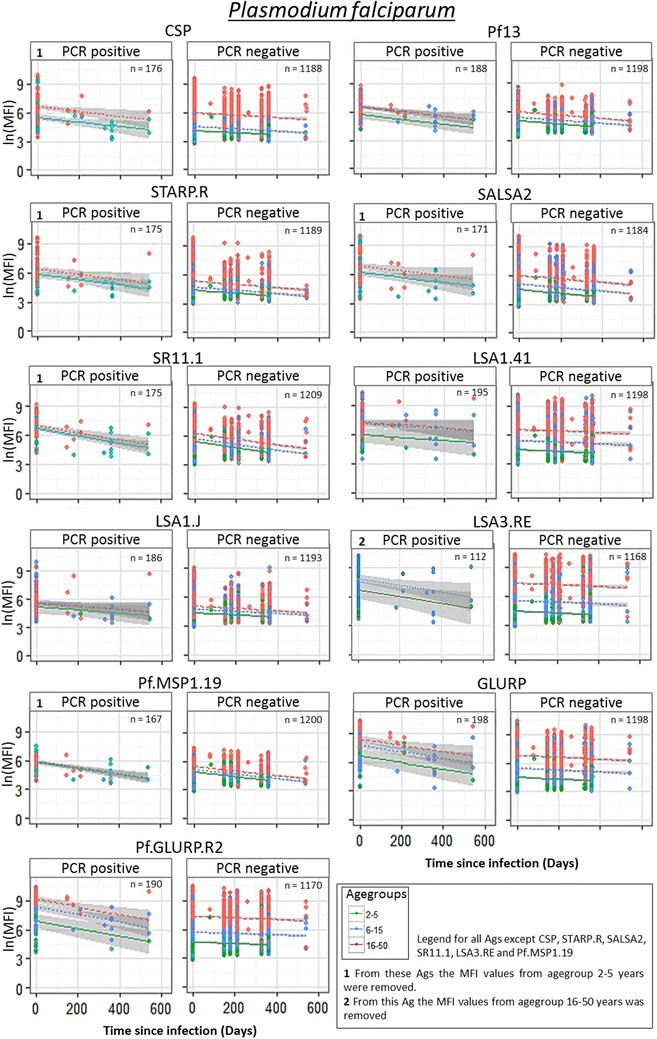
*Plasmodium falciparum* Ab profiles in relation to time since last infection compared to the systematically negative individuals. Each serological marker is analysed for *Plasmodium falciparum* infections and three age groups (1–5, 6–15 and 16–50). Samples collected through longitudinal follow-up were aligned, starting with putting the malaria episode detected by PCR at time point zero. Points represent the different samples in which a linear regression is drawn through with the 95% confidence intervals. These outcomes were then compared to the group of individuals being systematically negative over time, to see if the Ab-levels follow a similar or different decay over time between the parasite positive and negative individuals

**Fig. 4:**
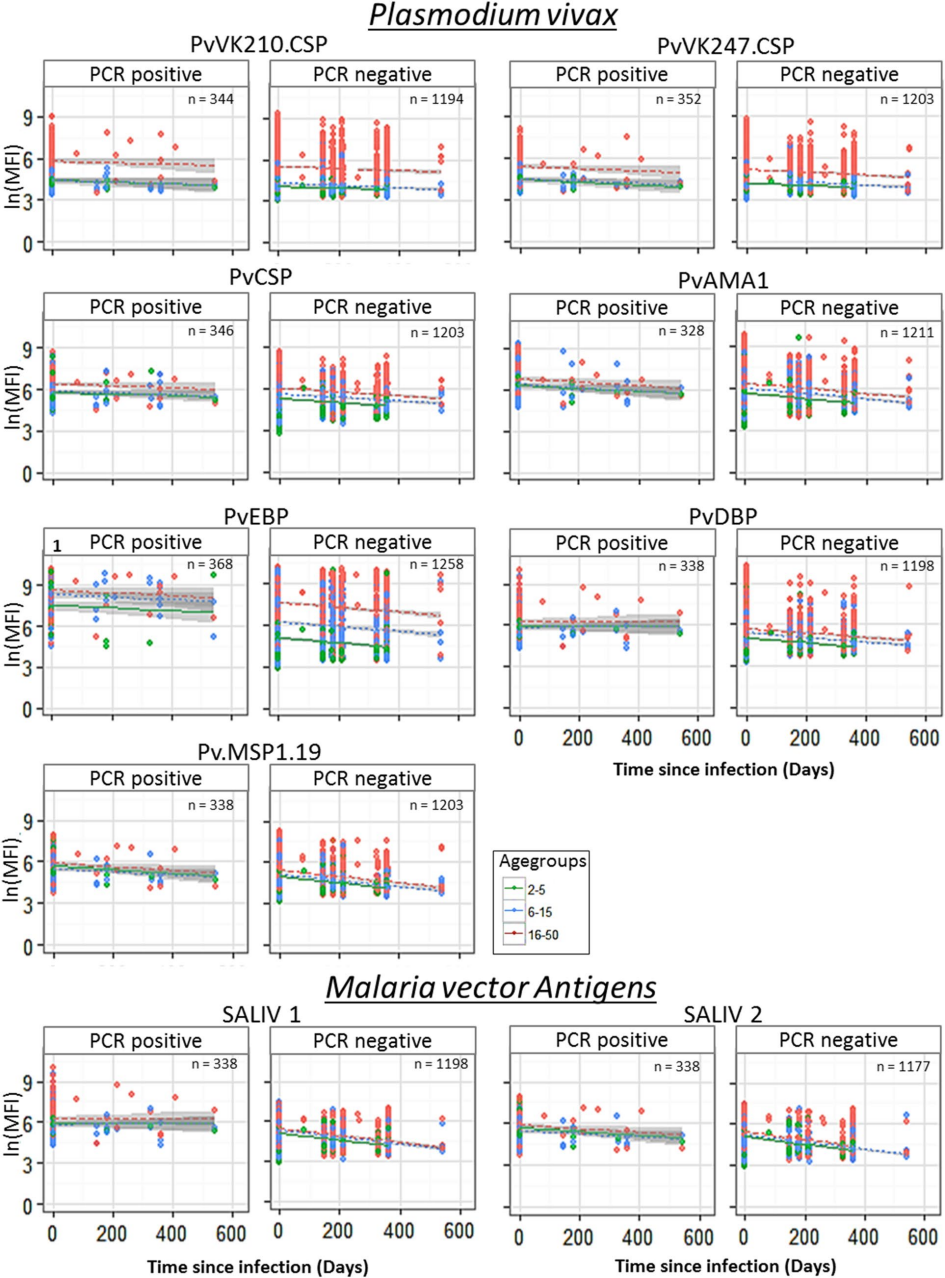
*Plasmodium vivax* Ab profiles in relation to time since last infection compares to the systematically negative individuals over time. Each serological marker is analysed for *Plasmodium vivax* infections and three age groups (1–5, 6–15 and 16–50). Samples collected through longitudinal follow-up were aligned, starting with putting the malaria episode detected by PCR at time point zero. Points represent the different samples in which a linear regression is drawn through with the 95% confidence intervals. These outcomes were then compared to the group of individuals being systematically negative over time, to see if the Ab-levels follow a similar or different decay over time between the parasite positive and negative individuals

**Fig. 5:**
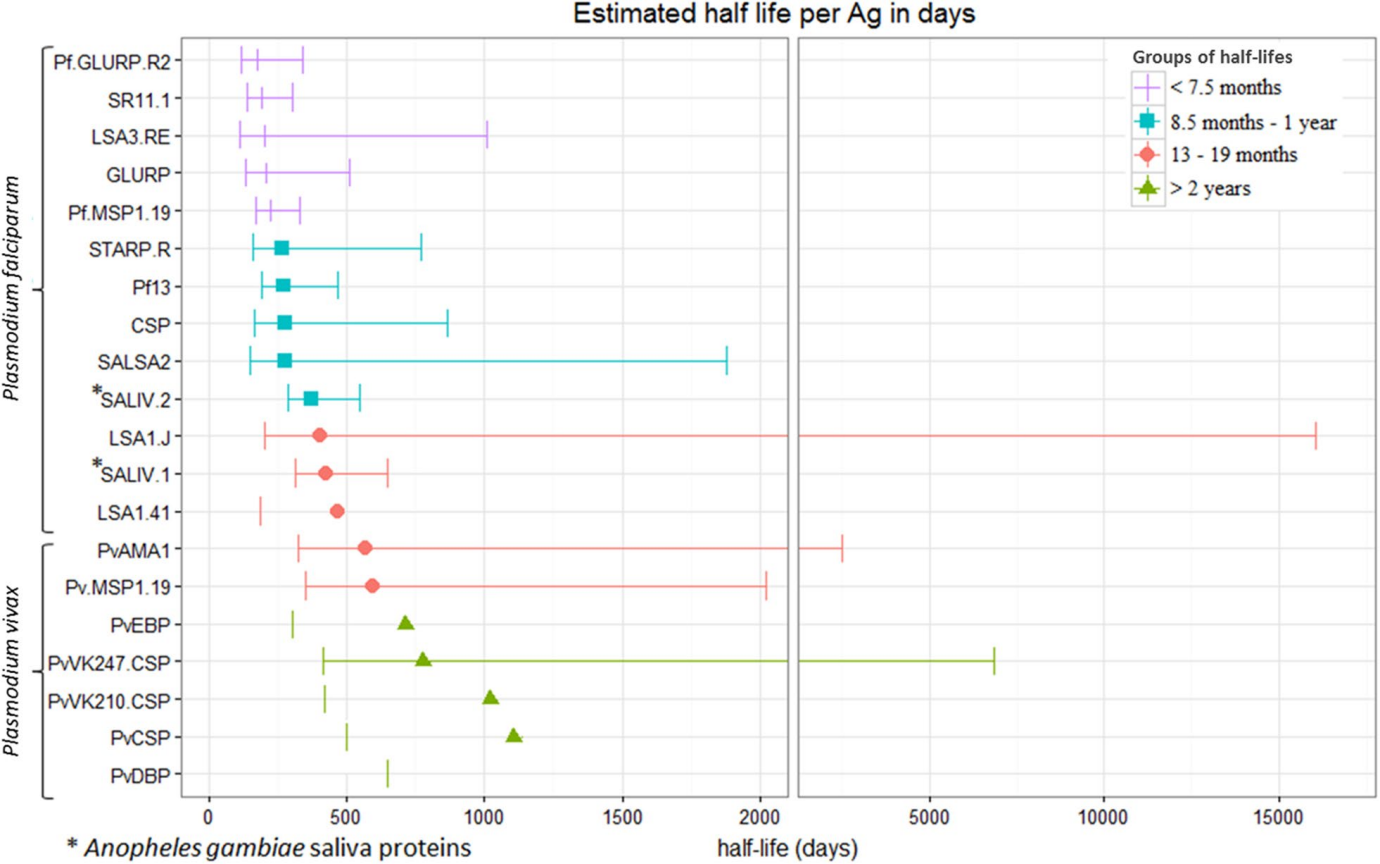
Forest plot representing the half-life per Ag in days. The half-lives based on the repeated measurement samples were estimated in days. A linear regression model was fitted on log-transformed MFI data taking into account age as factor. Estimated slopes and their 95% confidence intervals were used to obtain the half-life in days ($\text{t}_{1/2} = \frac{{ - { \ln }(2)}}{\lambda }$). This forest plot represents the half-lives of each Ag ordered from the shortest to the longest half-life. The *purple crosses* represent an estimated half-life shorter than 7.5 months, the *blue squares* range from 8.5 months until 1 year, the *red dots* range from 13 until 19 months, and the *green triangles* represent an estimated half-life longer than 2 years. The *error bars* are the 95% confidence intervals. A remarkable founding in this figure is that *P. falciparum* shows a clear difference between the short and long lived Ab-responses, while for the *P. vivax* only long lived Ab-responses are seen

There is a lot of variation between the estimated half-life per serological marker, suggesting that it is possible to distinguish short (seasonal) and long-term (year round) transmission trends. The model with time since infection and age groups as dependent variables (without interaction) was selected as the best model for all 20 Ags, suggesting that age does not have an effect on the slope of the Ab decay, although the Ab titres at time point zero differ between age groups (Figs. 3, 4). For nine antigens (CSP, STARP.R, SALSA2, SR11.1, LSA1.J, LSA3.RE, GLURP, Pf.GLURP.R2 and PvEBP) a pronounced decay of the Ab titres was observed (Fig. 3). When comparing these graphs with the group of individuals being systematically negative, Ags LSA3.RE, GLURP and Pf.GLURP.R2 showed a much steeper decay in the group of individuals being PCR positive one time. Besides, the graphs of the PCR positive seems to flow into the negative groups, showing a difference between the intercept of >2. This outcome suggests that these Ags are most promising for reflecting short-term patterns in malaria transmission. Estimated half-lives, ranging between 6 months (176 [CI 119–338] days) for Pf.GLURP.R2 and more than 2 years for some *P. vivax* Ags, clearly depend on the serological marker used (Fig. 5; Additional file 6). Abs against five Ags (Pf.GLURP.R2, SR11.1, LSA3.RE, GLURP and Pf.MSP1.19) show estimated half-life values shorter than approximately 7.5 months, with estimated half-lives ranging from 6 months (176 [CI 119–338] days) until ~7.5 months (225 [CI 171–329] days). Estimated half-lives of Abs against STARP.R, Pf13, CSP, SALSA2 and SALIV.2 range between ~8.5 months (263 [CI 159–768] days) until ~1 year (373 [CI 284–546] days) and of Abs against LSA1.J, SALIV.1, LSA1.41, PvAMA1 and PvMSP1.19 from ~1.1 year (402 [CI 203–16,035] days) until ~1.6 year (597 [CI 350–2020] days). Remaining Abs show an estimated half-life of more than 2 years.

For this analysis for some Ags (CSP, STARP.R, SALSA2, SR11.1, LSA1.41 and Pf.MSP1.19) samples from young children (2–5 years) were not included, due to a limited amount of samples available. For the same reason the age group of 16–50 years old was excluded for LSA3.RE.

### Intensity of the Ab-responses decreased together with the malaria exposure

To test whether Abs pick up recent malaria exposure in a population, the relation between Ab-titres (ln(MFI)) and the PCR prevalence of malaria in the province of Ratanakiri was explored. Therefore, the cluster PCR prevalence data from survey 2 and 4 was divided into different groups (forest plots: Additional file 7). In the forest plot, clusters with PCR prevalence between 3.5 and 7% were situated in the overlap between the medium- and high-level PCR prevalence and were not considered for present analysis, reflecting only those exhibiting high (7– >10%), medium (1–3.5%), and low (0– <1%) PCR-prevalence levels (Table 2). For Ags SALIV.1 and SALIV.2 all *Plasmodium* data (mono and mixed infections) were used, whereas the *P. falciparum* and *P. vivax* Ags were analysed species specific (only mono infections).

**Table 2:** Amount of cluster communities selected per species and per PCR prevalence level

PCR prevalence		*P. falciparum*	*P. vivax*	*Plasmodium*
High	S2	13	14	19
	S4	3	9	10
Medium	S2	18	26	24
	S4	21	23	24
Low	S2	52	39	26
	S4	61	44	31

Cluster communities were ordered per survey exhibiting high-, medium- and low-levels of PCR prevalence. This was performed on PCR data from all *Plasmodium* species (mono and mixed infections), *P. falciparum* (mono infections) and *P. vivax* (mono infections)

**Fig. 6:**
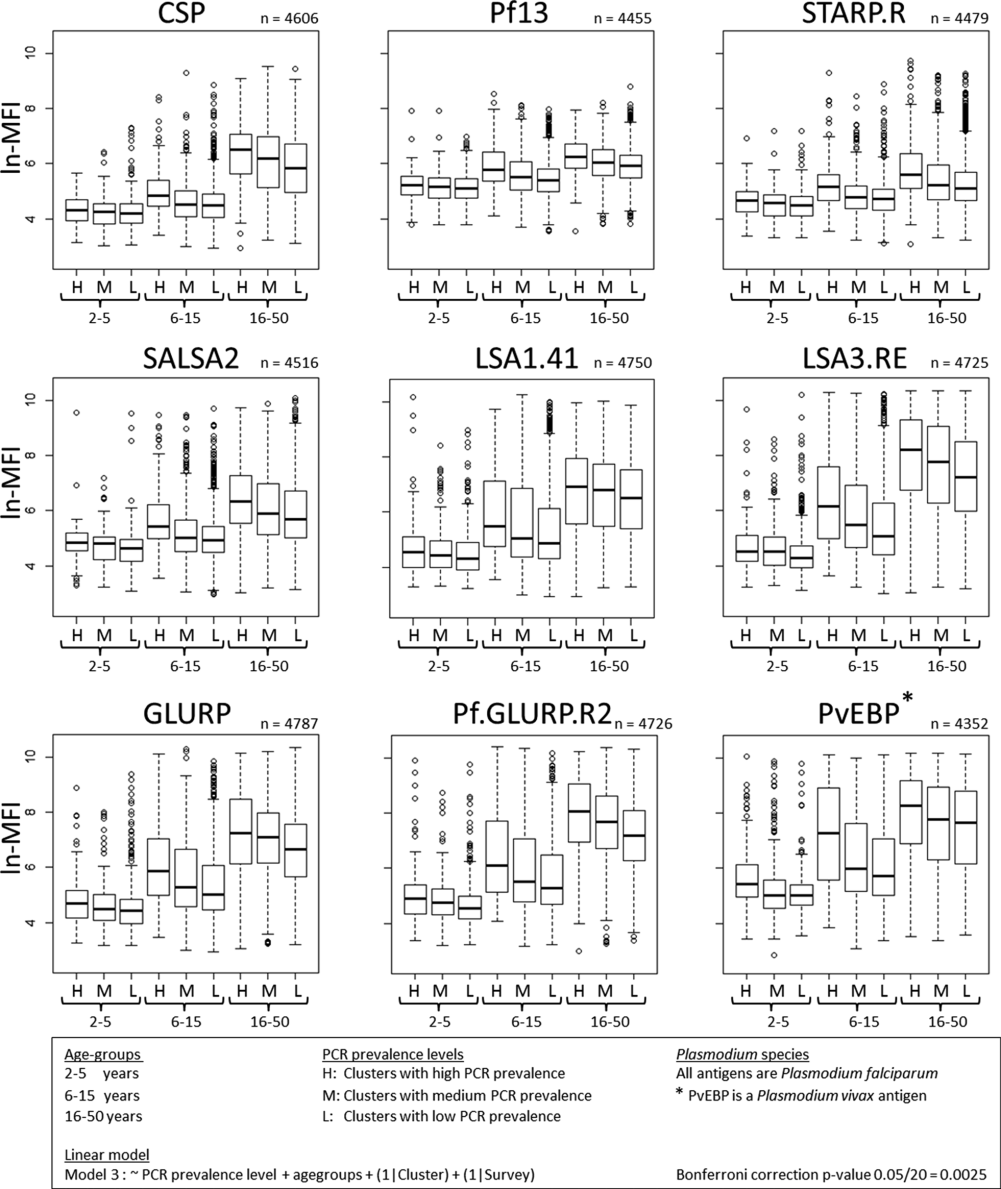
Ab-responses to *Plasmodium* Ags in relation to the risk of malaria exposure in different villages. To examine the associations between the Ab-levels and the malaria exposure clusters of villages were divided into three groups (those with high—(7– >10%), medium—(1–3.5%), and low—(0– <1%) prevalence levels). In addition, because age plays a major role in the analysis of Ab-responses, the three age groups (2–5, 6–15 and 16–50 years old) were included. Boxplots represent the medians, interquartile ranges and *error bars* show 95% confidence intervals. *Circles* represent outlier values. Ags showed to be statistical significant for model 3, a model with three groups of villages and age groups as a dependent variable (without interaction) taking into account Cluster and Survey as random effects. The figure only represents the Ags with the steepest decay (slope < −0.6)

For all 20 Ags, the model with PCR prevalence levels and age groups as dependent variables (without interaction) was selected. This model shows that when the PCR prevalence in groups of villages becomes lower, MFI levels also decrease (P < 0.0025; Additional file 8), within all age groups. Moreover, with decreasing endemicity Ab responses to the Ags presented in Fig. 6 show a more marked decline (drop in IRR between PCR prevalence level in respect to the reference group), as well as an increase in IRR with age. Biggest decline in IRR between PCR prevalence level was seen for Ags LSA3.RE, GLURP, Pf.GLURP.R2 and PvEBP, whereas the highest increase in IRR with respect to age was seen for Ags CSP, LSA1.41, LSA3.RE, GLURP, Pf.GLURP.R2 and PvEBP compared to the other Ags (Fig. 6).

### Selection of informative antigens for recent infection are based on three criteria

To determine which Ags best reflect recent malaria exposure (seasonal) the following three criteria were used: (1) the outcome of the estimated Ab-responses based on the differences in ln(MFI) between PCR positive and PCR negative individuals, (2) the assessed half-lives and (3) the estimated Ab-responses in relation to high-, medium- and low levels of *Plasmodium* exposure (Table 3). Based on these criteria the Ags that are most likely to reflect recent malaria exposure to *P. falciparum* are LSA3.RE, GLURP and Pf.GLURP.R2. Among the seven Ags tested for *P. vivax*, PvEBP is the most prominent choice.

**Table 3:** Three criteria to select the most promising antigens

	Antigens	Ags selection based on the three criteria			
		PCR^+^ vs PCR^−^ values^a^	Half-lives (PCR^+^)^b^	Differences in intercept^c^	Sensitivity by endemicity^d^
*P. falciparum*	CSP	+	+	+	+
	Pf13	+	+	−	+
	STARP.R	++	+	+	+
	SALSA2	+	++	+	+
	SR11.1	+	+	+	+
	LSA1.41	++	+	−	++
	LSA1.J	+	+	+	±
	LSA3.RE	++	++	++	++
	Pf.MSP1.19	+	−	−	−
	GLURP	++	++	++	++
	Pf.GLURP.R2	++	++	++	++
*P. vivax*	PvVK210.CSP	−	−	−	±
	PvVK247.CSP	−	−	−	±
	PvCSP	−	−	−	–
	PvAMA1	−	−	−	±
	PvEBP	++	−	++	++
	PvDBP	−	−	−	−
	Pv.MSP1.19	−	−	−	−
Vectors	SALIV1	−	−	−	−
	SALIV2	−	+	−	−

Three criteria are used to select the most promising Ags for recent malaria infection. These criteria are based on: (1) the outcome of the estimated Ab-responses based on the differences in ln(MFI) between PCR positive and PCR negative individuals, (2) the assessed half-lives with the best Ab-responses of <7.5 months followed by Ab-responses between 8.5 months until 1 year and finally (3) the sensitivity to the level of malaria endemicity in communities

^a^Difference in ln(MFI) (ln-MFI PCR+ −ln-MFI PCR−); ++ (value > 1), + (value > 0.5),−(value < 0.5)

^b^Half-lives estimated via linear regression models; ++ (half-life < 7.5 months), + (half-life between 8.5 months–1 year),− (half-life > 1 year)

^c^Difference in intercept between PCR+ and PCR− for half-life estimation; ++ (intercept difference > 2), + (intercept difference between 1 and 2), − (intercept difference < 1)

^d^Sensitivity by endemicity estimated via linear regression models; ++ (IRR of PCR prev. decline with > 0.2, IRR increase by age with >4), + (IRR of PCR prev. decline with >0.1, IRR increase by age with >1)

## Discussion

Several research groups recently developed multiplex serological assays for the detection of Abs against *Plasmodium* Ags [27, 38, 45–48]. Previous studies combining short- and long-lived markers for *Plasmodium* have generated knowledge about past and recent changes in malaria transmission within communities [13, 40, 49]. This has led to an increasing interest in the use of serology in acquiring epidemiological information that can be implemented in malaria control programmes [9, 13, 40, 49–55]. At this point, the majority of the studies and elimination programmes have focused in first instance on *P. falciparum* [13, 27, 38, 45, 46, 52, 56], which is easier to eliminate than *P. vivax* due to possible relapses in the latter one [57]. However, in contrast to other studies the current study was carried out in Southeast Asia (Cambodia) where all human malaria parasites are co-occurring [26, 58] and in which occupational and behavioural factors define the risk for malaria exposure [59–61]. As such, adolescents and mainly adults performing plantation work and forest activities are the main risk groups for malaria infection [59–62]. In this setting, insights into malaria transmission will be greatly enhanced by analysing age-adjusted Ab-responses directed against *Plasmodium* parasites including *P. vivax*. However, in this study age did not show any interactions on the outcomes.

The present study is to our knowledge the first study in Southeast Asia that uses multiplex serological measurements to document the half-life of IgG-Abs. Previous studies declare that Ab-levels follow a predictable pattern, in which every few months the Ab-level drops by half in the absence of re-infection [18, 41, 63]. To estimate this drop and apply those values in assessing the force of infection (FOI) in a study population, most researchers use a reversible catalytic conversion model [6, 40, 49, 51] on cross-sectional data. Such model is a simplification of a complex immunological process in which serological responses are converted to binary outcomes (seropositive or seronegative) through a threshold model [52]. Hence, these binary outcomes may lead to a loss of information due to the high range of MFI values within the seropositive group, and misclassification might occur because of subtle changes in the Ab-responses over time due to other infections or small laboratory variations [9]. Another limitation is that this model assumes a fixed sero-reversion rate, independent of age and transmission rate [51]. Therefore, some studies elaborated this model by comparing longitudinal- and cross-sectional data, which has led to large discrepancies in seroconversion and seroreversion rates [52]. Alternatively, linear regression models are used to provide information about the antibody acquisition [13, 21]. Helb et al. [13] and Yman et al. [12] estimated recent *P. falciparum* exposure at individual (longitudinal data) and population level (cross-sectional data). Studies based on longitudinal data mainly use approaches such as linear regression models [13, 20], generalized linear models (GLM) [64], or Ab-intensity models [12] to analyse Ab profiles.

A first criteria for selecting serological markers indicative for recent infection, is that the marker can at least pick up current infection. Therefore, the Ab intensity in *Plasmodium* infected people was compared to non-infected people. In agreement with results of previous studies, the present study shows that most Ab-responses (17/20) within each age group rise similarly by an infection. In contrast, in infected individuals Ab-responses to STARP.R, LSA1.41, GLURP, Pf.GLURP.R2 (among persons between 6 and 50 years) and PvEBP (among children from 2 to 15 years) seem to be influenced by age. [65]. The differences among children can be explained based on the role of immunological maturity-status [6], as children that acquire a malaria infection have the ability to boost their IgG titres, after which these Ab-titres decay again [66]. In this analysis it is important to take into consideration that the group of PCR-negatives is very large, and that PCR negative persons might have been infected in the six months between sampling periods. Moreover, for the PCR positive individuals, it is unknown when the infection was acquired.

In a next step, antibody decay rates (or half-lives) were estimated by using linear regression models that were fit on Ab-responses obtained from a cohort at different time points. In summary, the obtained results showed that the used serological markers could be divided in four groups with a wide variety of half-life estimates (Fig. 1; Table 3). Even though, limited information on the Abs persistence is available, some studies have previously estimated Ab half-lives [18, 20]. A study performed by Drakeley et al. [40] found a very long half-life of 49.8 years for Abs against Pv.MSP1.19, and Ondigo et al. [67] found half-lives from <1 year (3 Ags) to moderate (5–20 years for 3 Ags) and very long (>40 years for 5 Ags) by means of the threshold approach. Wipasa et al. [18] and Fowkes et al. [20] found a clearance of approximately 7.6 years [18] and 0.8–7.6 years [20], respectively, through linear regression models for the same Abs. The estimated Ab-clearance against merozoite Ags in the present study are best comparable with the latter results. However, these studies [18, 20] showed large ranges in 95% confidence intervals, which was also seen in the present study. The half-life estimates are in contrast with the underlying immunology showing the short period that merozoites need to reinvade erythrocytes. This is the short timespan in which the Abs have direct contact with the merozoite surface proteins, as this is the moment they are actually visible in the blood circulation [68]. A short clearance of Abs against merozoite Ags was also shown by Kinyanjui et al. [41] who estimated a clearance of 6–10 days and White et al. [66] that found a half-life of 7–72 days in children and 3–9 years for IgG Abs against AMA1, MSP1, MSP2 and CSP in infants, with a linear regression models. All these different outcomes suggest that using different model approaches (reversible catalytic conversion model and linear regression model) provide different outcomes, whereas with the linear regression models shorter half-lives were obtained. Moreover, large discrepancies in 95% confidence intervals were seen for some sporozoite related Ags as well. For these serological markers lower MFI values were observed [data not shown], resulting in larger 95% confidence intervals of Ab half-lives. This is not surprising as small numbers of sporozoites are only briefly present in the blood circulation to stimulate a sufficient production of sporozoite Abs, particularly, when situated in a region with low malaria transmission as the Mekong Subregion [17, 19]. Therefore, it has been previously suggested that the use of serological markers based on the sporozoite stage of malaria parasites might lead to an underestimation of the malaria endemicity in low transmission areas, while they possibly are more sensitive in hyper- and holo-endemic settings [19].

Finally, the cross-sectional data were examined to investigate whether the serological markers were capable of picking up current differences in malaria prevalence. Overall, for a lower PCR prevalence in a group of villages, the observed MFI levels were also lower within all age groups. This trend was similar as what was observed by Ambrosino et al. [27], who observed a rise in malaria specific Ab-responses with an increasing malaria exposure per village [27].

Based on the three different criteria i.e. (1) sensitive to infection in individuals, (2) sensitive to the level of endemicity in communities, and (3) a short half-life of IgG-Abs, the best serological markers (LSA3.RE, GLURP and Pf.GLURP.R2) that reflect recent *P. falciparum* exposure were identified. Moreover, Ags, CSP, Pf13, STARP.R, SALSA2, SR11.1, LSA1.41 and LSA1.J also seemed to respond well to a lesser extent. Therefore, these Ags should not be ruled out for future research in different settings. PvEBP was selected as the better candidate for *P. vivax* surveillance.

## Conclusion

Given the broad utility of serology, identifying serological markers for recent exposure seems a worthwhile investment [13]. In summary, it appears that for *P. falciparum* LSA3.RE, GLURP and Pf.GLURP.R2 are most likely to be reflective for recent exposure, whereas PvEBP is the only Ag from *P. vivax* that responds reasonably well, in spite of a half-life of more than 1 year. Therefore, it is essential to explore other Ags from *P. vivax*. The only available *P. malariae* antigen did not provide a good response. It is remarkable that the best reflective Ags are mainly liver- and blood stage Ags, whereas in contrast to high endemic areas sporozoite level Ags are not useful in this setting (low-endemic). It is worth noting that all 20 Ags exhibit a direct link with the endemicity. In short, these Ags should certainly not be excluded for further in-depth analyses related to malaria control programmes. Another important aspect is that the use of Ab intensity data rather than dichotomizing the continuous Ab-titre data (positive vs negative) will lead to an improved approach for serological surveillance.

## Additional files

**Additional file 1.** Raw data file. This file contains all raw data used for all analyses in this study.

**Additional file 2.** Example of Levey Jenning Charts plotted for the quality control of the immunoassay used for screening the field bloodspot samples. Data analysis started with a quality control on the ΔMFI-values of the 100% positive control pool samples (A). The dots represent each positive control sera sample in duplicate per plate. If these dots fell out of the −2SD and +2SD (red area), these plates were rejected and re-analysed. The same quality control was also performed on the percentage positivity ($\frac{\Delta MFI Low positive control (Ag1)}{\Delta MFI High positive control (Ag1)}$ × 100%) calculated from the 50% positive control pool samples per Ag (B). Based on the outcome of both graphs, 30 plates were rejected and reanalysed.

**Additional file 3.** Multiplex assay on a dilution series of the control samples to confirm the linear range of the assay. The mean MFI values of the control samples (per dilution and per Ag) were plotted. Graphs were made species specific. The vertical black lines show the spot where the 1:100, 1:400 and 1:1600 dilution is situated. Each of the Ags follow the linear range of the assay.

**Additional file 4.** Example of quantile regression model utilized on a MA-plot to detect differences between the duplicate samples. The quantile regression model is a way to validate samples and their duplicates on consistency. The red and black dots represent the samples and duplicates of which the red dots represent the outliers. The dotted lines represent upper and lower fences (Q3 + 1.5IQR and Q1−1.5IQR), where Q1 is the lower 25th quantile and Q3 the upper 25th quantile and IQR = Q3 − Q1. The outer solid lines represent lower and upper bounds that classify the difference between the outliers and non-outliers.

**Additional file 5.** Example of a histogram in which the MFI values are plotted against different age categories. Given the trend between the various ages it has been observed that this might be an important factor that has to be taken into account. The boxplots represent the medians, interquartile ranges and error bars (95% confidence intervals) per age. Circles represent outlier values. In the first plot is performed on all 8,654 samples, while the second plot has been performed on only the PCR positive samples. Looking at the trend it is clear that the MFI increase according to the age. Samples above the age of 50 show variation in MFI levels and were therefore removed from the analysis. Therefore age groups from 2–5, 6–15 and 16–50 years are chosen.

**Additional file 6.** Estimates of the half-life per serological marker. The half-lives based on the repeated measurement samples were estimated in days. A linear regression model was fitted on log-transformed MFI data taking into account age as factor. Estimated slopes and their 95% confidence intervals were used to obtain the half-life in days (${\text{t}}_{1/2} = \frac{{ - { \ln }(2)}}{\lambda }$).

**Additional file 7.** Example of a Forest plot created to define the different levels of Clusters according to the PCR prevalence. To analyse Abs that pick up recent changes in malaria transmission, forest plots were created on the cluster prevalence data from survey 2 and 4 per *Plasmodium* species (mono infections). Each cluster was examined twice, once for survey 2 and once for survey 4. The dots represent the measured PCR prevalence (% proportion) for a certain village. The error bars represent the 95% confidence intervals. The villages are ordered from the village with the lowest to the village with the highest PCR % proportion. Different groups are automatically selected with a R-software [28] package. The group that showed a high overlap between two different levels of prevalence was removed for further analysis.

**Additional file 8.** Incidence rate ratios and p values estimated to indicate difference between the Ab intensity (ln(MFI)) and the levels of exposure per age group (6–5 and 16–50 years). Significant differences between the different levels of exposure (High-, Medium-, Low prevalence) were seen for all specific Ags (p<0.00025). The incidence rate ratios (IRR) confirm that the MFI value decline together with the PCR prevalence in the villages and rise with age. Incidence Rate Ratio that indicates for how much (if > 1) or less (if < 1) the covariates decline with PCR prevalence level or rise with an increasing age. This is performed in respect to the reference category and LCI and UCI representing the lower and upper 95% confidence intervals based on the total sample size of n = 6,499 individuals from 98 villages.
