# Supplementary figures and images for: Serological markers to measure recent changes in malaria at population level in Cambodia
Source: Malar J. 2016 Nov 4;15:529. doi: 10.1186/s12936-016-1576-z (PMC5096337; doi:10.1186/s12936-016-1576-z)

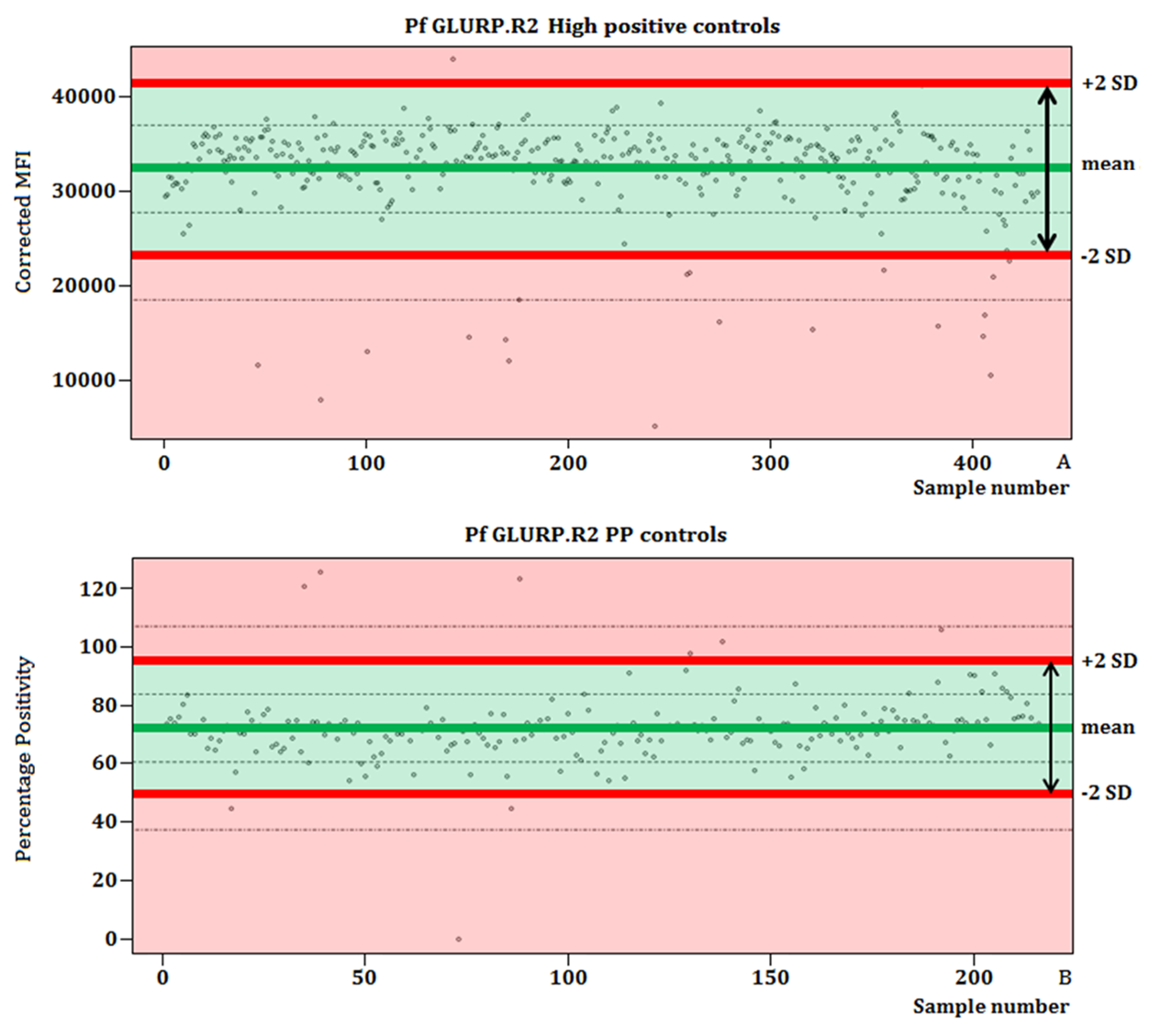

Supplement: Supplementary file 2 — Additional file 2. Example of Levey Jenning Charts plotted for the quality control of the immunoassay used for screening the field bloodspot samples. Data analysis started with a quality control on the ΔMFI-values of the 100% positive control pool samples (A). The dots represent each positive control sera sample in duplicate per plate. If these dots fell out of the −2SD and +2SD (red area), these plates were rejected and re-analysed. The same quality control was also performed on the percentage positivity ($\frac{\Delta MFI Low positive control (Ag1)}{\Delta MFI High positive control (Ag1)}$ΔMFILowpositivecontrol(Ag1)ΔMFIHighpositivecontrol(Ag1) × 100%) calculated from the 50% positive control pool samples per Ag (B). Based on the outcome of both graphs, 30 plates were rejected and reanalysed. [file 12936_2016_1576_MOESM2_ESM.png]

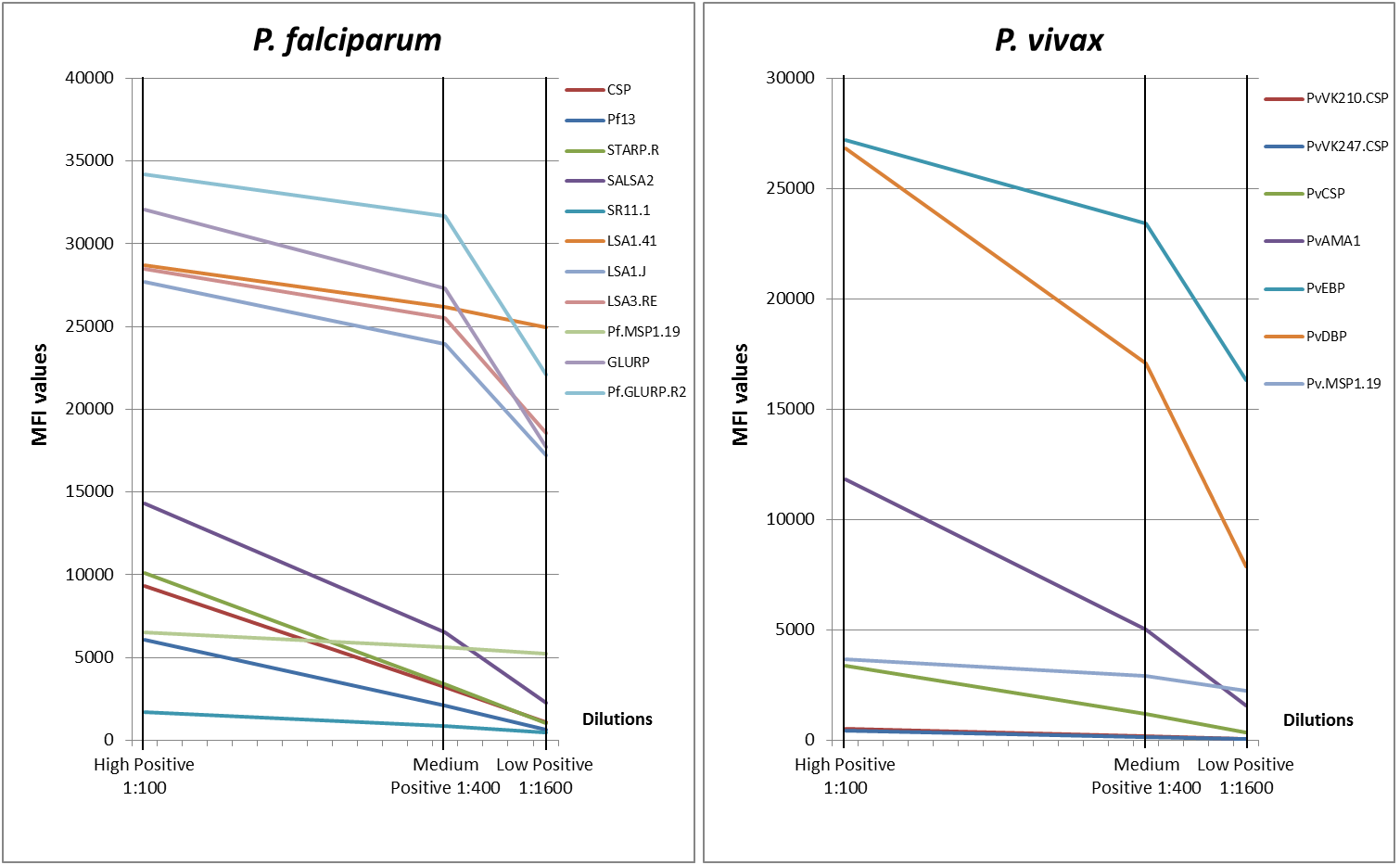

Supplement: Supplementary file 3 — Additional file 3. Multiplex assay on a dilution series of the control samples to confirm the linear range of the assay. The mean MFI values of the control samples (per dilution and per Ag) were plotted. Graphs were made species specific. The vertical black lines show the spot where the 1:100, 1:400 and 1:1600 dilution is situated. Each of the Ags follow the linear range of the assay. [file 12936_2016_1576_MOESM3_ESM.png]

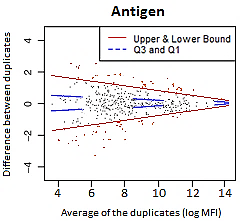

Supplement: Supplementary file 4 — Additional file 4. Example of quantile regression model utilized on a MA-plot to detect differences between the duplicate samples. The quantile regression model is a way to validate samples and their duplicates on consistency. The red and black dots represent the samples and duplicates of which the red dots represent the outliers. The dotted lines represent upper and lower fences (Q3 + 1.5IQR and Q1−1.5IQR), where Q1 is the lower 25th quantile and Q3 the upper 25th quantile and IQR = Q3 − Q1. The outer solid lines represent lower and upper bounds that classify the difference between the outliers and non-outliers. [file 12936_2016_1576_MOESM4_ESM.png]

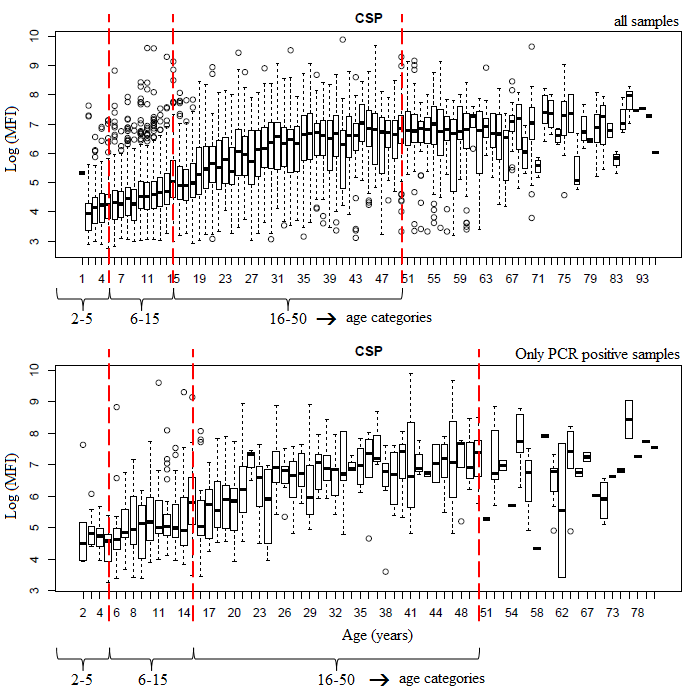

Supplement: Supplementary file 5 — Additional file 5. Example of a histogram in which the MFI values are plotted against different age categories. Given the trend between the various ages it has been observed that this might be an important factor that has to be taken into account. The boxplots represent the medians, interquartile ranges and error bars (95% confidence intervals) per age. Circles represent outlier values. In the first plot is performed on all 8,654 samples, while the second plot has been performed on only the PCR positive samples. Looking at the trend it is clear that the MFI increase according to the age. Samples above the age of 50 show variation in MFI levels and were therefore removed from the analysis. Therefore age groups from 2–5, 6–15 and 16–50 years are chosen. [file 12936_2016_1576_MOESM5_ESM.png]

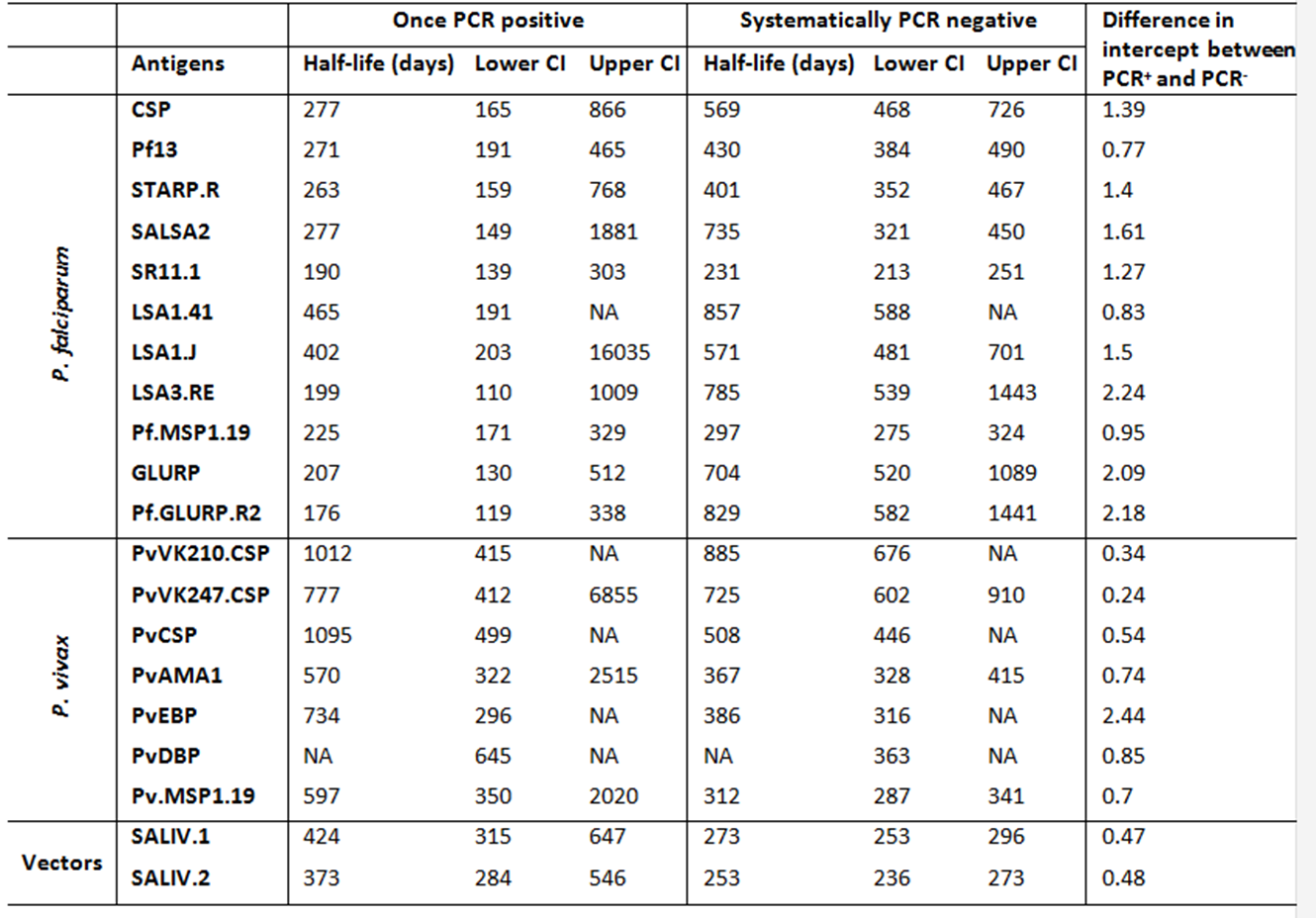

Supplement: Supplementary file 6 — Additional file 6. Estimates of the half-life per serological marker. The half-lives based on the repeated measurement samples were estimated in days. A linear regression model was fitted on log-transformed MFI data taking into account age as factor. Estimated slopes and their 95% confidence intervals were used to obtain the half-life in days (${\text{t}}_{1/2} = \frac{{ - { \ln }(2)}}{\lambda }$t1/2=-ln(2)λ). [file 12936_2016_1576_MOESM6_ESM.png]

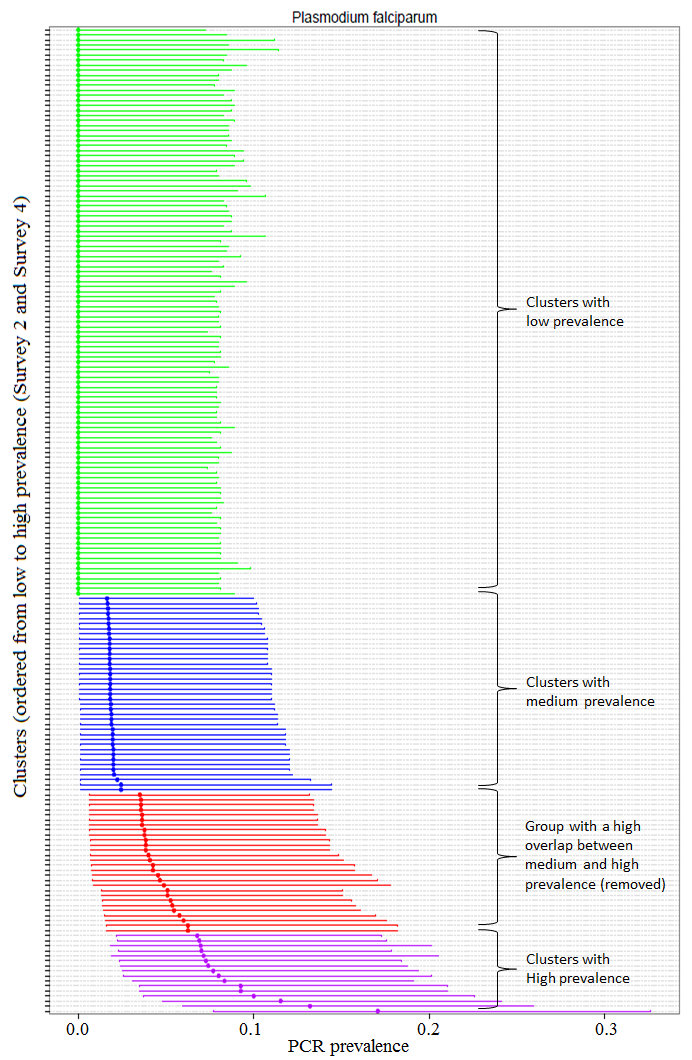

Supplement: Supplementary file 7 — Additional file 7. Example of a Forest plot created to define the different levels of Clusters according to the PCR prevalence. To analyse Abs that pick up recent changes in malaria transmission, forest plots were created on the cluster prevalence data from survey 2 and 4 per Plasmodium species (mono infections). Each cluster was examined twice, once for survey 2 and once for survey 4. The dots represent the measured PCR prevalence (% proportion) for a certain village. The error bars represent the 95% confidence intervals. The villages are ordered from the village with the lowest to the village with the highest PCR % proportion. Different groups are automatically selected with a R-software [28] package. The group that showed a high overlap between two different levels of prevalence was removed for further analysis. [file 12936_2016_1576_MOESM7_ESM.png]
